# *Toona sinensis* Leaf Aqueous Extract Improves the Functions of Sperm and Testes via Regulating Testicular Proteins in Rats under Oxidative Stress

**DOI:** 10.1155/2012/681328

**Published:** 2012-10-08

**Authors:** Bu-Chin Yu, Wen-Jen Yu, Chun-Yung Huang, Ying-Hsin Chen, Yun-Ching Tsai, Chen-Chin Chang, Sue-Joan Chang

**Affiliations:** ^1^Department of Life Sciences, National Cheng Kung University, No. 1, University Road, Tainan 701, Taiwan; ^2^Department of Biotechnology, The University of Hong Kong, Taichung 433, Taiwan; ^3^Department of Seafood Science, National Kaohsiung Marine University, Kaohsiung 811, Taiwan; ^4^Department of Living Science, Tainan University of Technology, Tainan 710, Taiwan

## Abstract

*Toona sinensis* leaf (TSL) is commonly used as a vegetable and in spice in Asia. In this study, feeding with aqueous extract of TSL (TSL-A) alleviated oxidative stress and recovered the motility and functions of sperm in rats under oxidative stress. Protein expressions in testes identified by proteomic analysis and verified by Western blot demonstrated that TSL-A not only downregulated the level of glutathione transferase mu6 (antioxidant system), heat shock protein 90 kDa-**β** (protein misfolding repairing system), cofilin 2 (spermatogenesis), and cyclophilin A (apoptosis) but also upregulated crease3-hydroxy-3-methylglutaryl-coenzyme A synthase 2 (steroidogenesis), heat shock glycoprotein 96, and pancreatic trypsin 1 (sperm-oocyte interaction). These results indicate that TSL-A promotes the functions of sperm and testes via regulating multiple testicular proteins in rats under oxidative stress, suggesting that TSL-A is a valuable functional food supplement to improve functions of sperm and testes for males under oxidative stress.

## 1. Introduction

Excess production of reactive oxygen species (ROS) has been associated with the male infertility [1]. The accumulation of ROS impaired sperm and testes functions including impairment of spermatogenesis, suppression of steroidogenesis, and disruption of sperm-oocyte interaction through lipid peroxidation and DNA damages [2]. In addition, ROS-induced oxidative damage in mitochondrial proteins, leading to the collapse of mitochondrial membrane potential (MMP) and loss of sperm motility, has been documented [3]. The benefits of antioxidant s [4] and herbal medicine, such as Ginseng [5] and *Astragalus* [6], have been reported on the sperm quality, sperm-oocyte interaction, and fertilization. However, the choice of dietary supplements to assist in male reproductive disorders is limited.

The leaf of *Toona sinensis *(TSL) is extensively used as a vegetable and in spice in Asia. The TSL aqueous extracts (TSL-A) are the mixtures of polyphenols and flavonoids with various bioactivities, such as antioxidant properties [7]. Our previous studies have demonstrated that TSL-A exhibited the antioxidant activity in rats under oxidative stress [8] and protected human spermatozoa against oxidative damage, indicating a regulatory function in sperm and testes [9]. However, the mechanism of TSL-A on the sperm and testes functions has yet to be fully elucidated. 

In the present study, rats under oxidative stress induced by injection of H_2_O_2_ for 2 weeks and fed with TSL-A and gallic acid (GA), a major component in TSL-A [7], for 8 weeks were investigated for the underlying mechanism. Factors influenced sperm functions, including ROS, MMP, and motility of sperm, were measured by flow cytometry and microscope, respectively. Proteomic analysis provides a better understanding of dynamic and overall views of the cell machinery under various conditions. Thus, the differentially expressed proteins in testes of rats fed with TSL-A were identified by proteomic analysis and confirmed by Western blot.

## 2. Material and Methods

### 2.1. Reagents and Materials

The 2-DE reagent including acrylamide solution (25%) thiourea, immobiline dry strips, immobilized pH gradients (IPG) buffer (pH = 3–10), IPG cover mineral oil, iodoacetamide (IAA), TEMED, trifluoroacetic acid (TFA), 2-DE clean-up kit, 2-DE Quant kit, and silver staining kit were purchased from GE healthcare (Piscataway, NJ, USA). 3-[(3-cholamidoropyl) dimethylammonio]-1propane sulfonate (CHAPS), dithiothreitol (DTT), EDTA, NaF, NaCl, NP-40, PMSF, sodium dodecyl sulfate (SDS), Tween 20, urea, Na_3_VO_4_, methanol (HPLC grade, >99.9%), and sodium deoxycholate were purchased from Sigma-Aldrich (St. Louis, MO, USA). Protein marker and polyvinylidene fluoride (PVDF) membrane were purchased from Invitrogen **(**Carlsbad, CA, USA**)**. trisbase and trichloroacetic acid (TCA) were purchased from J. T. Baker (Phillipsburg, NJ, USA). The primary and secondary antibodies for Western blotting were purchased from Santa Cruz **(**Santa Cruz, CA, USA). 

### 2.2. Animals and Treatments

Six-week-old male Sprague-Dawley (SD) rats were purchased from the laboratory animal center of National Cheng Kung University, Tainan, Taiwan, and animal experiments were approved by the Institutional Animal Care and Use Committee (IACUC, Approval no. 96152). Animals were housed individually in a well-ventilated room maintained at 22 ± 2°C and 12 h light-dark cycle. The oxidative stress in the animals was induced by intraperitoneal (i.p.) injection with H_2_O_2_, 1 mmol/kg body weight (b.w.) every other day for 2 weeks before the start of experiment, and continued for next 8 weeks. After the 2 weeks of oxidative induction, rats were randomly divided into five groups: vehicle group, H_2_O_2_ group, H_2_O_2_ treatment only, TSL-A group, the H_2_O_2_ treatment plus TSL-A (13 mg/kg b.w/day) and gallic acid group, and H_2_O_2_ treatment plus gallic acid (100 mg/kg b.w/day) feeding. According to our previous study [8], we used the optimal dose of gallic acid plus H_2_O_2_ group as a positive control in the present study. Animals were sacrificed with CO_2_ at the end-point of this experiment, and then testes were isolated immediately and stored at −80°C for further analyses. 

### 2.3. Preparation and Fractionation of TSL Extracts

TSL-extracted powder purchased from Taiwan Toona Biotech Corporation (Kaohsiung, Taiwan) was dissolved in 99.5% ethanol and centrifuged at 3000 rpm at 4°C for 12 min. The pellet was lyophilized and further dissolved in serial ethanol for the serial extractions to obtain TSL-A which was employed in the present study to investigate its effect on the testicular protein expression.

### 2.4. Measurement of Sperm ROS, MMP Levels, and Sperm Motility

Spermatozoa were collected from the caput, corpus, and cauda epididymis by mincing the tissue in PBS containing glucose (5 mmol/L) and protease inhibitors and then purified by washing through a Percoll step gradient (20% : 30% : 40% in PBS) by centrifugation at 250 g for 10 minutes [10]. Recovered spermatozoa were either used for sperm motion, ROS, or MMP analysis. A Beckman-Coulter Epics XL-MCL Analyzer (Beckman-Coulter, Hialeah, FL) equipped with a single 488 nm excitation source was used for all flow cytometric analyses. The intracellular ROS was measured by detecting intracellular DCFH2-DA oxidation in spermatozoa adapted from [11, 12]. The acetate moiety is cleaved by cellular esterases, leaving impermeant, nonfluorescent, 2′,7′-dichlorodihydrofluorescein (DCFH). The DCFH is oxidized by H_2_O_2_ to dichlorofluorescein (DCF), which emits fluorescence at 530 nm in response to 488 nm excitation. The increase in DCFH2-DA oxidation was measured as an increase in FL1 (green fluorescence, 525 nm) on a log scale for 5000–10,000 events. DiOC6, a lipophilic, cationic, fluorescent probe, can bind to mitochondrial inner membrane and be used for quantitative measurement of MMP [13]. Rat sperms were incubated with 4 nmol/L DiOC_6_ at 37°C for 15 min. The level of MMP was measured as an increase in FL1 on a log scale for 5000–10,000 events. The progressive sperm motility was evaluated as described by Sönmez et al. [14]. For this purpose, a slide was placed on microscope stage and allowed to warm to a temperature of 37°C by means of a heater stage. Several droplets of trisbuffer solution were then dropped onto the slide, and a very small droplet of fluid obtained from left cauda epididymis with a pipette was dropped on the Tris buffer solution and covered with a cover slip. The percentage of motile sperm was determined in Makler sperm counting chambers (Sefi-Medical Instrument, Haifa, Israel), by using a light microscope at a magnification of 400x. During spermatozoa activation, immotile sperm cells (ISCs) were counted, and when the activation gets stopped, whole sperm cells (WSCs) were counted in per microscopic area by naked eyes, and then motile sperm cells (MC) were calculated as MC = WSC−SC. Motility was determined as the percentage of sperm actively moving forward according to that formula: motility %(M) = motile sperm/whole sperm × 100.

### 2.5. Protein Preparation for Two-Dimensional Gel Electrophoresis

Testes removed from the sacrificed rats were homogenized in lysis buffer (8 mol/L urea, 4% CHAPS, 40 mmol/L trisbase, 1% DTT, and 0.5% IPG buffer) at 4°C for 1 h. The homogenate was centrifuged at 7,500 ×g, 4°C for 30 min. After the centrifugation, 2D Clean-up kit was utilized to precipitate proteins according to manufacturer's protocols. The dried protein sample was dissolved in rehydration buffer (8 mmol/L Urea, 2% CHAPS, 0.5% IPG buffer, 0.002% bromophenol blue, and 20 mmol/L DTT) and stored at −80°C. Protein concentration was determined by 2D Quant Kit. After the protein quantification, the samples were stored at −80°C for further analyses.

### 2.6. Two-Dimensional Gel Electrophoresis and Silver Stain

2-DE was performed using an IPGphoreTM isoelectric focusing (IEF) system and an Ettan DALTsix Electrophoresis unit (GE healthcare, Piscataway, NJ, USA). Protein sample (140 *μ*g) was loaded onto an immobilized pH 3–10 linear gradient strip (24 cm), followed by rehydration for 16 h. IEF was then performed at 25°C in the following manner: 250 V for 2 h, 500 V for 2 h, 1000 V for 2 h, 4000 V for 2 h, and 8000 V for 800000 Vhr. At the end of IEF, the IPG strips were equilibrated with 65 mmol/L DTT in equilibration buffer (6 mol/L urea, 2% w/v SDS, 30% v/v glycerol, 0.002% bromophenol blue, and 50 mmol/L Tris, pH 8.8) for 15 min and subsequently equilibrated with 135 mmol/L IAA in equilibration buffer for another 15 min. After the equilibration, the IPG strips were immediately placed on the top of a 12% SDS-PAGE (1.5 mm, 20 × 24 cm). The second dimension gels were then overlaid with molten 0.5% agarose solution in SDS electrophoresis buffer. Electrophoresis was performed at 20°C, starting at 100 V for 30 min, followed by 17 w per gel until the dye reached the bottom of the gels (~5 h). 2-DE was run in two types of experiment to minimize individual variation in type 1 and to decrease the technique deviation in type 2 [15]. In type 1, identical amounts of protein from each animal in one group were pooled to run triplicate gels. In type 2, one gel was run for each individual animal in one group. Protein spots appeared on all three triplicates gels in type 1 and at least three out of four individual gels in type 2 experiment and expressed differentially more than 2-fold between groups were selected.

### 2.7. In-Gel Digestion

Protein spots were excised manually and transferred into siliconized 0.5 mL eppendorf tubes. All in-gel digestions of proteins were performed manually with trypsin (Promega Crop., Madison, WI, USA) in a laminar flow hood with disposable gloves to reduce the keratin contamination. The gel pieces were washed twice with 50% v/v ACN and 50% v/v ACN/25 mmol/L ammonium bicarbonate and placed at 56°C for 45 min in 10 mmol/L DTT and 55 mmol/L iodoacetamide in 25 mmol/L ammonium bicarbonate to be reduced and alkylated. Approximate 10 *μ*L of 0.1 g/L of modified trypsin digestion buffer in 25 mmol/L ammonium bicarbonate was added to the gel pieces, and the gel pieces were incubated overnight at 37°C. After the centrifugal and removing the supernatant, the peptides were further extracted from the gel piece by incubating in 50% v/v ACN/5% v/v formic acid. The selected peptides were added with 20 *μ*L of 5% v/v ACN/0.1% v/v formic acid and subjected to MS analysis for protein identification.

### 2.8. RP-Nano-HPLC-ESI-MS/MS and Database Search

RP-nano-HPLC-ESI-MS/MS was performed to identify 2-DE separated proteins. The In-gel tryptic digest for protein was fractionated using a C18 microcapillary column (75 *μ*m i.d. × 15 cm) at a flow rate of 200 nL/min with a nano-HPLC system (LC Packings) coupled to an ion trap mass spectrometer (LCQ DECA XP Plus, ThermoFinnigan) equipped with an ESI source. The HPLC system consists of a micropump/UV detection module (UltiMate, Dionex, Amsterdam, The Netherlands), a column switching module (Switchos, Dionex, Amsterdam, The Netherlands), and an autosampler module (Famos, Dionex, Amsterdam, The Netherlands). The elution solutions used were 5% v/v ACN/0.1% v/v formic acid (buffer A) and 80% v/v ACN/0.1% v/v formic acid (buffer B). Chromatographic elution was performed using a 40 min solvent gradient from 0 to 60% buffer B. As peptides eluted from the microcapillary column, they were electrosprayed into the ESI-MS/MS with the application of a distal 1.3 kV spraying voltage. Each cycle of one full scan mass spectrum (*m/z* 150–2000) was followed by three data-dependent MS/MS spectra. After data acquisition, the files were searched by querying the Swiss-Prot database or NCBI database or both using MASCOT (http://www.matrixscience.com/). In brief, all. dta files generated from each respective LC-MS/MS data set were manually merged into one merge.txt file by merge.exe and subjected to database searching. The RAW files were analyzed with Biowork3.1 (Thermo Electron Corp.) as the peak list-generating software. The peak list was used to query the Swiss-Prot database using the MASCOT program with the following parameters: peptide mass tolerance, 1 Da; MS/MS ion mass tolerance, 1 Da; enzyme set as trypsin and allowance up to two missed cleavages; variable modifications considered were carboxymethyl (C, K), deamidation (N, Q), oxidation (M), and pyroglu (D, E); peptide charge, 2+ and 3+; taxonomy limited to Rattus, number of protein entries searched, 40,029. The mass lists were used for protein identification in the NCBInr 20061215 (4,255,399 sequences; 1,462,302,728 residues) nonredundant protein database by Mascot 2.0 search engine (MATRIX SCIENCE Inc., London, UK). The following acceptance criteria were used: individual ions scores of Mascot search results above the cutoff score of 40 indicate identity or extensive homology (considered significant: over 95% probability that the result is not false positive) and at least three matching peptides.

### 2.9. Western Blotting

Testes were ultrasonic cell-break and lysed in lysis buffer (20 mmol/L Tris, 150 mmol/L NaCl, 1 mmol/L EDTA, 1% NP-40, 1 mmol/L NaF, 1 mmol/L Na_3_VO_4_, and 1 mmol/L PMSF) at 4°C for 1 h. Protein concentrations were determined by BCA protein assay kit (Thermo, MA, USA). Aliquots of tissue extracts containing equal amounts of protein were separated by SDS-PAGE on 10–15% gels for different molecular weight range, and then proteins were transferred to PVDF membranes and blocked by rocking for 1 h at room temperature in blocking buffer (phosphate-buffered saline (PBS) with 0.1% Tween 20 (PBST) and 5% nonfat dry milk). Blots were incubated with primary antibody overnight at 4°C, washed with PBST for three times, treated with secondary antibody for 1 h at room temperature, and washed with PBST for three times. Signals were detected with an enhanced chemiluminescence kit according to the manufacture's protocol (Millipore, Bedford, MA, USA) and analyzed using Fuji Multi Gauge software (FUJIFILM, Tokyo, Japan).

### 2.10. Statistics

Data were analyzed using the statistics software package SAS (version 8 e; SAS Inst. Inc., Cary, NC, USA). Duncan's test was performed for the analysis of variance on the various experimental groups. Data were presented as means ± SEM. Differences were considered to be significant if the calculated probability of their occurring by chance was less than 5% (*P* < 0.05).

## 3. Results and Discussion

**Figure 1:**
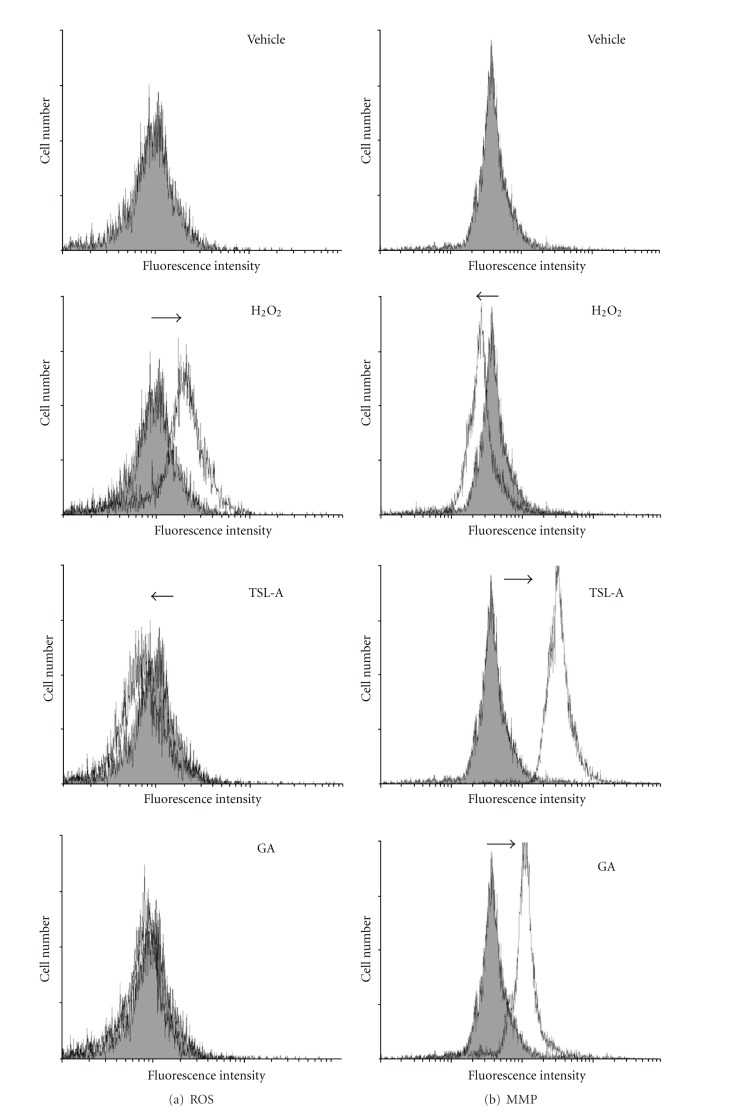

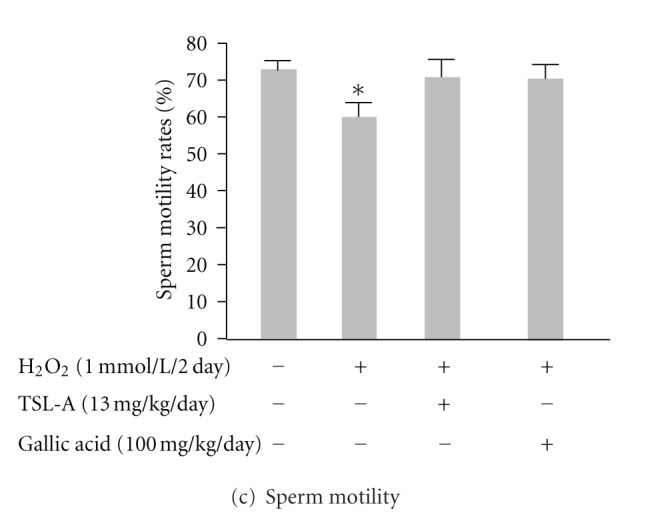
Changes of the levels of sperm ROS, MMP, and motility in rats under oxidative stress without and with TSL-A or GA feeding. (a) Change of ROS level in sperm. Sperms from different group were incubated with DCFH2-DA as an indicator for ROS; (b) change of the MMP level in sperm. Sperms from different group were incubated with DiCO6 as an indicator for MMP; (c) sperm motility of different group. Rats were randomly divided into five groups: vehicle group, H_2_O_2_ group, the H_2_O_2_ treatment plus TSL-A feeding (TSL-A), H_2_O_2_ treatment, and H_2_O_2_ treatment plus GA feeding (GA). Both the ROS and MMP levels of sperm were measured by flow cytometry. The sperm motility was determined by microscope. The symbols “→” and “←” represent the increased and decreased ROS and MMP level compared with that of vehicle group, respectively. *P < 0.05 compared with vehicle group.

**Figure 2:**
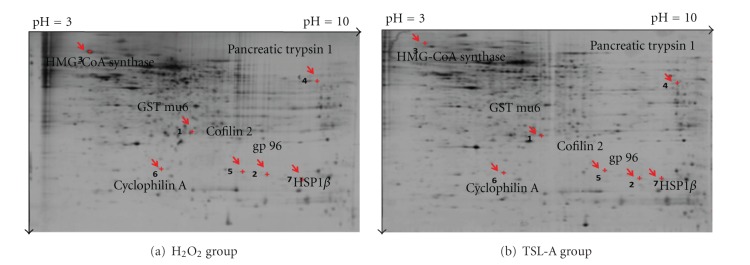
2-DE gel analysis of protein expression in testes of rats treated with H_2_O_2_ and H_2_O_2_ plus TSL-A. Protein expressions in testes of SD rats IP injected with 1 mmol H_2_O_2_/kg b.w every other day (a) and fed with normal diet, or TSL-A (13 mg/kg b.w/day) (b) for 8 weeks, were separated by 2-DE. Protein spots with numbers represented the seven-protein expression more than 2-fold analyzed by the ImageMaster 2D Platinum Software. Details of the proteins were given in Table 1.

Administration with TSL-A and GA decreased ROS (Figure 1(a)), increased MMP (Figure 1(b)), and restored sperm motility (Figure 1(c)) in sperms of rats under oxidative stress. Seven testicular proteins detected by proteomic analysis were regulated by TSL-A, including glutathione transferase mu6 (GST mu6), heat shock protein 90 kDa-*β* (HSP-1*β*), 3-hydroxy-3-methylglutaryl-Coenzyme A synthase 2 (HMG CoA synthase), pancreatic trypsin 1, heat shock glycoprotein 96 (gp96), cofilin 2, and cyclophilin A (Table 1 and Figure 2). Results of Western blot indicated that the levels of proteins (gp96, HMG-CoA synthase, and pancreatic trypsin 1) which decreased by H_2_O_2_ were found to be significantly increased by TSL-A when compared with normal group (Figure 2(b)). In contrast, proteins (GST mu6, HSP-1*β*, cofilin 2, and cyclophilin A) whose levels increased by H_2_O_2_ were significantly decreased by TSL-A when compared with normal group (Figure 2(b)). GA decreased levels of HSP-1*β*, cofilin 2, and cyclophilin A that were also significantly decreased by TSL-A. Among those proteins (gp96, HMG-CoA synthase, and pancreatic trypsin 1) whose levels were significantly increased by TSL-A, GA significantly increased level of HMG-CoA synthase and pancreatic trypsin 1 (Figure 2(b)).

The antioxidant properties of TSL-A may contribute to maintaining the mitochondrial function leading to the improvement of sperm motility (Figure 1). Beyond the antioxidant properties of TSL-A, proteomic results demonstrated that GST mu6, HSP-1*β*, cofilin 2, and cyclophilin A were downregulated, and HMG-CoA synthase, gp96, and pancreatic trypsin 1were upregulated by administration of TSL-A in testes of rats under oxidative stress. TSL-A reduced the increased levels of GST mu6, HSP-1*β*, cofilin 2, and cyclophilin A protein expression by H_2_O_2_ (Figure 2) indicating that the endogenous antioxidant system (GST mu6) [16], misfolding repairing system (HSP-1*β*) [17], spermatogenesis (cofilin 2) [18], and cell apoptosis (cyclophilin A) [19] in testes were protected by TSL-A under oxidative stress. 

GST mu6 is a member of GST family and mainly expressed in testes [16]. The increased levels of GST mu6 protein expression by H_2_O_2_ were repressed by the presence of TLS-A to normal level (Figure 2), suggesting that antioxidant activity [7, 8] in TSL-A may effectively react with ROS prior to the initiation of oxidative damage [20], resulting in the nearly normal expression of GST mu6 protein.

HSPs are a class of functionally related proteins whose expression is increased when cells are exposed to pathologic conditions [17, 20]. HSPs have been reported to increase the activity of antioxidant and prevent the misfolding of antioxidant enzymes, resulting in the reduction of oxidative damage in animal and cell models [17]. The oxidative stress-induced HSP-1*β* expressions for preventing the aggregation of misfolding protein was decreased significantly by supplement of vitamin E in HepG2 cells [17]. In the present study, TSL-A repressed the increased HSP-1*β* protein by H_2_O_2_ to normal level (Figure 2) indicating that the underlying mechanism by TSL-A may be attributed to its antioxidant activity and similar to that by vitamin E in stopping the chain reaction of the oxidative stress [17, 20]. 

**Figure 3:**
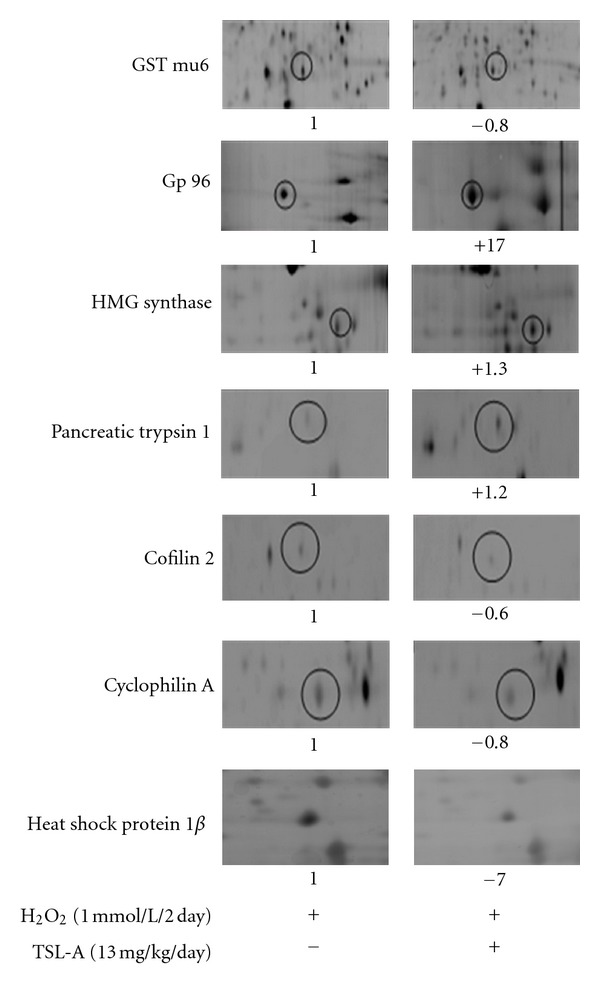
Alterations of 7 identified protein expressions from 2-DE gel. Protein expressions in testes of SD rats IP injected with 1 mmol H_2_O_2_/kg b.w every other day and fed with normal diet and TSL-A (13 mg/kg b.w/day) for 8 weeks were separated by 2-DE. Protein spots with numbers represented the 7-protein expression more than 2-fold analyzed by the ImageMaster 2D Platinum Software. Details of the proteins were given in Table 1. The symbols “+” and “−” represent the upregulation and downregulation of the protein expression, respectively. The expression fold was that of the H_2_O_2_ group.

Cofilin, a family of actin-binding proteins which disassemble actin filaments, is widely distributed throughout nonmuscle (cofilin 1) and muscle (cofilin 2) cells [18]. H_2_O_2_-induced abnormal cofilin aggregation in the mitochondria is suggested to contribute to the loss of MMP, failure of spermatogenesis, and spermatocytes apoptosis [18]. The elevated levels of sperm MMP by TSL-A (Figure 1(b)), in combined with alleviation of abnormal H_2_O_2_-induced cofilin 2 expressions by TSL-A (Figure 3) demonstrated that TSL-A prevented the abnormal aggregation of cofilin 2, maintained the sperm MMP and spermatogenesis, and inhibited the cell apoptosis in testes of rats under oxidative stress.

Previous study has demonstrated that germ cells in rat testis contain relatively high levels of cyclophilin A protein expression which is associated with cell apoptosis [19]. In the present study, protein expressions of cyclophilin A were significantly induced by H_2_O_2_ (Figure 3) indicating that protein expression of cyclophilin A was elevated for H_2_O_2_-induced cell apoptosis. The increased levels of cyclophilin A protein expressions were repressed to normal level by TSL-A (Figure 3) indicating that TSL-A prevents the H_2_O_2_-induced cell apoptosis. 

In the present study, TSL-A upregulated gp96, HMG-CoA synthase, and pancreatic trypsin 1 which were suppressed by H_2_O_2_, indicating that TSL-A is beneficial for sperm-oocyte interaction (gp96 and pancreatic trypsin 1) and steroidogenesis (HMG-CoA synthase) under oxidative stress. 

Gp96 is an endoplasmic reticulum glycoprotein believed to function in the translocation of protein across the endoplasmic reticulum membrane and the folding of denature protein as well as in multimer assembly [21]. Gp96 was also reported to be involved in the sperm maturation and sperm-oocyte interaction [22]. In the present study, the repressed levels of gp96 protein expression by H_2_O_2_ were increased by the presence of TSL-A (Figure 3) which indicated that the administration of TSL-A restores the sperm motility and maturation. 

HMG-CoA synthase in human gonads is one of the rate-limiting enzymes in the synthesis of sex hormone, such as testosterone [23]. The increased ROS level has been reported to be associated with the decreasing HMG-CoA synthase expression resulting in the decline of steroidogenesis and testosterone concentration [24]. The activities of enzymes in steroidogenesis were found to be recovered by vitamin C in testes [25]. The HMG-CoA synthase protein expression which increased significantly by TSL-A in this study may contribute to the maintenance of steroidogenesis.

Trypsin is an essential factor in spermatogenesis and fertilization [26]. In the present study, the reduced protein expression of pancreatic trypsin 1 by H_2_O_2_ was elevated significantly by TSL-A (Figure 3) suggesting the protection of TSL-A on the spermatogenesis and fertilization in rats under oxidative stress. 

**Figure 4:**
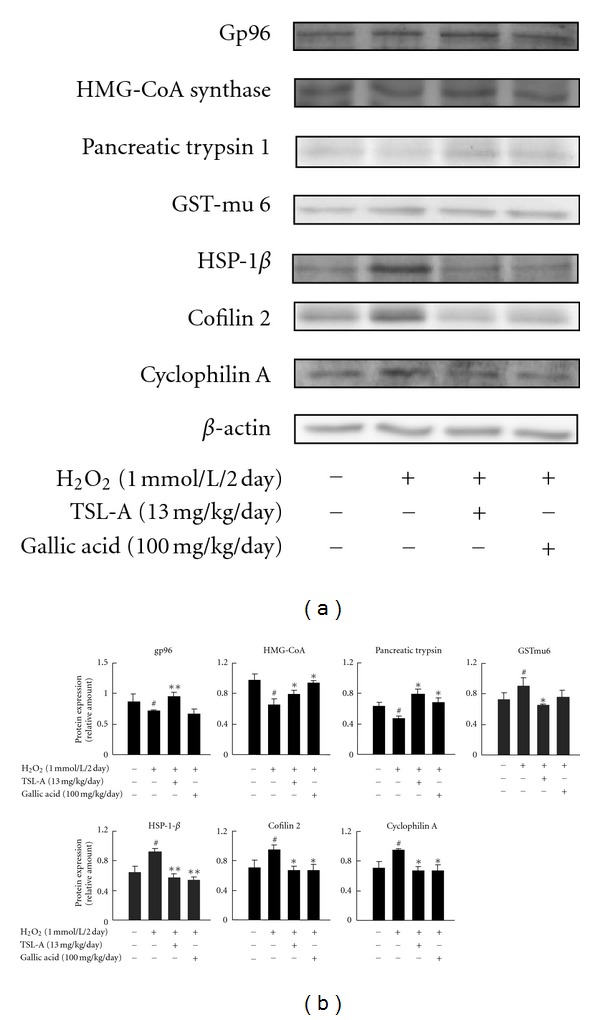
Confirmation of identified protein expressions by Western blot. (a) Expressions of identified proteins in testes of rats under oxidative stress and fed with normal chow, TSL-A (13 mg/kg/day), and GA (100 mg/kg/day) for 8 weeks were analyzed by Western blot; (b) quantification of identified proteins. All values are mean ± SEM (*n* = 4). ^#^ *P* < 0.05 compared with control group. *P < 0.05 and **P < 0.01 compared with H_2_O_2_-treated groups.

The beneficial effects of vegetables have been proposed to be the synergistic combinations of phytochemicals in vegetables [27]. The major phytochemical in TSL-A, which is GA exhibiting its protection against oxidative damage [7], may contribute to the protective effects of TSL-A on the functions of sperm and testes. In this study, GA regulated protein expressions of HMG CoA synthase, pancreatic trypsin 1, HSP-1*β*, cofilin 2, and cyclophilin A by a similar trend of TSL-A (Figure 4). On the contrary, TSL-A-regulated gp96 and GST mu 6 protein expressions which were not affected by GA may be attributed to the other components besides GA in TSL-A (Figure 4). Therefore, our results demonstrated that the protection of TSL-A for testes and sperm functions in rats under oxidative stress was through the multiple bioactivities of various phytochemicals.

## 4. Conclusion

In conclusion, TSL-A repressed the ROS level, maintained the MMP, and restored the sperm motility in sperms of rats under oxidative stress. The protection of TSL-A is attributed to its regulation of proteins involved in not only the antioxidant activity, but also protein misfolding repairing system, spermatogenesis, steroidogenesis, sperm maturation, and sperm-oocyte interaction in testes of rats under oxidative stress. Thus, we suggest that TSL-A is a valuable functional food supplement to improve sperm quality and testes functions for males under oxidative stress.

## Figures and Tables

**Table 1:** Proteins differentially expressed in testes of rats fed with H_2_O_2_ and H_2_O_2_ plus TSL-A and identified by RP-nano-HPLC-ESI-MS/MS.

Spot no.	Identified protein	pI-MW(kD)	Sequence coverage	Queries matched	MASCOT score	Accession no.
1	Glutathione transferase mu6 (GST mu6)	5.99_25.6	38%	15	501	XP_575012
2	Heat shock glycoprotein 96 (gp 96)	5.02_74.1	17%	10	549	NP_001012197
3	3-Hydroxy-3-methylglutary coenzyme A synthase 2 (HMG CoA synthase)	8.86_56.8	4%	2	84	NP_775117
4	Pancreatic trypsin 1	4.71_25.9	8%	2	75	NP_036767
5	Cofilin 2	9.18_28.9	16%	2	157	XP_345675
6	Cyclophilin A	8.34_17.8	19%	2	113	NP_058797
7	Heat shock protein 1 b (HSP90 1*β*)	4.97_83.2	9%	5	236	NP_032328

## Abbreviations

GA: Gallic acid
Gp96: Heat shock glycoprotein 96
GST mu6: Glutathione transferase mu 6
HMG CoA synthase: 3-Hydroxy-3-methylglutaryl-Coenzyme A synthase 2
H_2_O_2_: Hydrogen peroxide
HSP1*β*: Heat shock protein 90 kDa-*β*
MMP: Mitochondrial membrane potentials
ROS: Reactive oxygen species
TSL: *Toona sinensis* Roem leaves.
